# White root rot of *Bletilla striata*: the pathogen, biological characterization, and fungicide screening

**DOI:** 10.3389/fmicb.2024.1374137

**Published:** 2024-06-03

**Authors:** Fang Liang, Xuejing Jiang, Lijuan Liu, Feihu Wang, Feng Liu, Saixue Hu, Lijun Tan, Xiao Chen, Yixuan Xu, Xiulan Xu, Liqiong Jiang, Yinggao Liu, Chunlin Yang

**Affiliations:** ^1^College of Forestry, Sichuan Agricultural University, Chengdu, China; ^2^National Forestry and Grassland Administration Key Laboratory of Forest Resources Conservation and Ecological Safety on the Upper Reaches of the Yangtze River & Forestry Ecological Engineering in the Upper Reaches of the Yangtze River Key Laboratory of Sichuan Province, College of Forestry, College of Forestry, Sichuan Agricultural University, Chengdu, China; ^3^Forestry Research Institute, Chengdu Academy of Agricultural and Forestry Sciences, Chengdu, China

**Keywords:** *Bletilla striata*, white root rot, *Fusarium solani*, biological characterization, fungicide screening

## Abstract

*Bletilla striata* is an endangered traditional medicinal herb in China. In May 2020, the emergence of white root rot severely impacted the quality and yield of *B. striata*, affecting about 5% of the plants at plant nurseries of the Chengdu Academy of Agricultural and Forestry Sciences. Through a series of experiments and evaluations, the pathogen was identified as *Fusarium solani*. This is the first report of *B. striata* white root rot caused by *F. solani* in Sichuan, China. To better understand this disease and provide data support for its control, a combination of morphological, molecular characterisation and pathogenicity determination was used in this study for assessment. Meanwhile, the effects of different carbon and nitrogen sources, culture medium, temperature, photoperiod and pH on mycelial growth and spore production of *F. solani* were investigated. In addition, effective fungicides were screened and the concentration ratios of fungicides were optimized using response surface methodology (RSM). The experimental results showed that sucrose was the optimum carbon source for the pathogen, and the optimum temperature and pH were 25°C and pH 7, respectively, while light did no significant effect. Effective fungicides were screened, among which difenoconazole showed the strongest inhibition with EC_50_ of 142.773 µg/mL. The optimum fungicide concentration scheme (difenoconazole, pyraclostrobin, and thiophanate-methyl at 395.42, 781.03, and 561.11 µg/mL, respectively) was obtained using response surface methodology (RSM) to improve the inhibition rate of 92.24 ± 0.34%. This study provides basic data for the pathogen characterization of *B. striata* white root rot and its potential fungicides in Sichuan, China. In addition, the optimal fungicide concentration ratios were obtained through response surface methodology (RSM) optimization, which significantly enhanced the fungicidal effect and provided a scientific basis for the future control of *B. striata* white root rot.

## 1 Introduction

*Bletilla striata* (Thunb. ex Murray) Rchb. f., a member of the Orchidaceae family, is a rare traditional Chinese medicinal herb with a variety of uses (Deng et al., 2020; Qian et al., 2021). Its use as a Chinese medicine was initially mentioned 2000 years ago in the *Divine Farmer’s Classic of Materia Medica*. *B. striata* tubers, which are rich in polysaccharides, benzyl compounds, terpenoids, and other chemicals (Ge et al., 2022), are typically used to treat a range of conditions due to their medicinal properties such as tumor resistance, cardiovascular disease prevention, hemostasis promotion, antioxidant activity, antifungal activity, and free radical scavenging function (Yu et al., 2016; Ge et al., 2022; Zhu, 2023). Wild *B. striata* in China is mainly distributed in the area south of the Qinling Mountains–Huaihe River, encompassing the provinces of Sichuan, Yunnan, Hubei, Hunan, Jiangxi, and Zhejiang (Li et al., 2017; Zhao et al., 2019). However, due to rising consumer demand, poor reproductive capacity, and slow growth, wild *B. striata* is now endangered (Wang, 2018).

In recent years, the area used for artificial cultivation has been increasing due to the growing consumer demand for *Bletilla striata* (Du, 2019). Due to the use of monocultures and non-standard pesticides, artificial cultivation is increasing the risk of disease in *B. striata*, which decreases its yield, quality, and ornamental value. For instance, a multitude of pathogens can induce leaf diseases, including leaf spot, which is caused by *Fusarium commune*, *F. asiaticum*, *F. ipomoeae*, *F. solani*, *F. avenaceum*, *Daldinia concentrica*, and *Epicoccum sorghinum* (Ke et al., 2018; Li et al., 2019; Song, 2019; Zhou et al., 2020; Wang H. Y. et al., 2022); leaf blight, which is caused by *Curvularia reesii* (Chen et al., 2023); anthracnose, which is caused by *Colletotrichum orchidophilum* and *Coll. fructicola* (Wang S. M. et al., 2022; Yang K. et al., 2022); southern blight, which is caused by *Sclerotium rolfsii* (Yu et al., 2018); rust, which is caused by *Coleosporium bletiae* (Xu et al., 2023); gray mold, which is caused by *Botrytis cinerea* (Li et al., 2018); and blight, which is caused by *C. bletiae* (Yuan et al., 2019). Additionally, two pathogens, *F. fujikuroi* and *Rhizoctonia* sp., can cause stem rot (Zeng et al., 2012; Chen et al., 2018). Moreover, *B. striata* is vulnerable to root rot, which can have a major negative impact on the yield and quality of medicinal ingredients. The root rot pathogens of *B. striata* reported so far include *Fusarium oxysporum, Epicoccum sorghinum*, and *Dactylonectria torresensis* (Sun et al., 2013; Zhou et al., 2018; Li et al., 2020).

In May 2022, we discovered a severe *Bletilla striata* white root rot in a nursery of the Chengdu Academy of Agriculture and Forestry Sciences in Sichuan Province. In the early stage of the infection, the leaves had wilted tips and brown lesions, which eventually became wet spots. In the middle stage of the infection, the roots became soft, rotten, and covered with white mycelia and brown necrotic leaf lesions expand, causing some leaves to wilt or fall off. Finally, the plant growth was significantly weakened, the vascular bundles, root cortex, and epidermis deteriorated, and the water transport function eventually disappeared. The roots were enveloped by numerous white mycelia, with the emergence of a white radial mycelium or rhizomorph extending from the root surface into the soil. The brown leaf lesions expanded to cover entire leaves and even young stems, leading to the desiccation and shedding of most of the leaves. Eventually, the entire plant dies. We found that the symptoms exhibited by this disease at the initial stage were quite similar to those of the reported leaf spot disease of *B. striata* (Zhou et al., 2019), and its root disease was similar to root rot, mainly causing severe root rot. Unlike the previous root rot diseases of *B. striata*, we found that the roots of this disease were covered by a large number of white mycelia, and there were also obvious rhizomorph in the soil. This distinctive feature is consistent with the symptoms of white root rot. However, to our knowledge, there are no reports on the identification and control of the causal agent of *B. striata* white root rot in China.

Thus, the main aim of this study was to determine the main pathogen causing *Bletilla striata* white root rot by conducting a pathogenicity test and morphological and molecular identification. Additional aims were to determine the pathogen’s biological characteristics (to gain basic information about its epidemiology), evaluate the pathogen’s reactions to key environmental variables, and screen for effective fungicides against the pathogen *in vitro*. The results provide an important reference for the future control of the disease.

## 2 Materials and methods

### 2.1 Site, sample collection and fungal isolation

A total of 10 *Bletilla striata* seedlings with severe disease were collected from two *B. striata* nurseries of the Chengdu Academy of Agriculture and Forestry Sciences (103°51′26.3412″ E, 30°42′11.7396″ N) in Chengdu, Sichuan Province, China. The root surface was washed with sterile distilled water three times (to remove surface dirt) and dried at room temperature. Pure cultures were obtained from single conidia on PDA plates based on the Chomnunti method (Chomnunti et al., 2014). They were then preserved until identification. Plant and pathogen specimens were deposited in the Herbarium of Sichuan Agricultural University (SICAU) and the Culture Collection of Sichuan Agricultural University (SICAUCC), China, respectively.

### 2.2 Morphological and molecular identification

Cultures were grown on PDA for 7 days, at 25°C, under 12 h light/12 h dark for recording growth rates, shape, texture and colour of the colonies. For morphological identification, the microscopic characteristics of the pathogen were observed. We observed microscopic characteristics, such as conidia pile, conidiophores, conidiogenous cells, conidia and number of septa, using an Olympus BX43 (Olympus in the Japanese). No fewer than 50 measurements were made for each feature using the Image Frame Work (IFW 0.9.0.7).

The New Plant Genomic DNA Kit (Beijing Aidlab Biotechnologies Co., Ltd., Beijing, China) was used to extract genomic DNA from the fresh fungal mycelium of two representative isolates (SICAUCC 23-0086 and SICAUCC 23-0087). In Fusarium systematics, through NCBI comparison and morphological characteristics description, fungi in this study belongs to the *Fusarium solani* species complex (Xie et al., 2022). Subsequently, polymerase chain reaction (PCR) amplification was performed for the ribosomal RNA internal transcribed spacer (rDNA-ITS), translation elongation factor 1-alpha region (*tef*1-α), and RNA polymerase II subunit B (*rpb*2) (Gräfenhan et al., 2011; Wanasinghe et al., 2021). The primer pairs are listed in Table 1. Polymerase chain reaction (PCR) was performed in 25 μL reaction mixture containing 22 μL Master Mix (Beijing LABLEADBiotech Co., Ltd., Beijing, China), 1 μL DNA template and 1 μL each of forward and reverse (10 μM) primers. The amplification reactions were performed as described by White et al. (1990), O’Donnell et al. (1998), and Liu et al. (1999). PCR products were sequenced at Hangzhou Youkang Biotech Co., Ltd., Chengdu, China. Based on BLAST searches in GenBank and recent publications (O’Donnell et al., 2012; Lombard et al., 2015; Xu et al., 2020), using the ITS, rpb2, and *tef*1-α and sequence data, reference sequences were downloaded and separate phylogenetic analyses, based on single gene datasets were carried out to initially determine the placement of the species. Information on the taxa used and GenBank accession numbers of our novel species are listed in Table 2. Alignments for the individual locus matrices were generated with the online version of MAFFT version 7.429 (Katoh et al., 2019) and ambiguous regions were excluded using BioEdit version 7.0.5.3 (Hall, 2004). Phylogenetic analyses of *Fusarium solani* were performed using ITS, rpb2, and *tef*1-α dataset (Vaidya et al., 2011), and rooted by *Fusarium illudens* NRRL 22090. A maximum likelihood (ML) phylogenetic tree was constructed as described by Xu et al. (2020). The tree was constructed and edited using FigTree v1.4.2.

**Table 1:** Gene markers and primer pairs were used in this study.

Gene markers	Primers	Sequences of primers 5′–3′	References
ITS	ITS5	GGAAGTAAAAGTCGTAACAACG	White et al. (1990)
	ITS4	TCCTCCGCTTATTGATATGC	
*tef*1-α	EF1	ATGGGTAAGGARGACAAGAC	O’Donnell et al. (1998)
	EF2	GGARGTACCAGTSATCATG	
rpb2	*rpb*2-7cf	ATGGGYAARCAAGCYATGGG	Liu et al. (1999)
	*rpb*2-11ar	GCRTGGATCTTRTCRTCSACC	

**Table 2:** Specimen information and GenBank accession numbers of the sequences used in this study.

Species	Isolates	GenBank accession nos.		
		ITS	rpb2	*tef*1-α
*F. ambrosium*	NRRL 20438	AF178397	JX171584	AF178332
*F. bataticola*	NRRL 22402	AF178408	FJ240381	AF178344
*F. bostrycoides*	NRRL 31169	DQ094396	EU329564	DQ246923
*F. brasiliense*	NRRL 31757	EF408514	EU329565	EF408409
*F. breve*	VG157	MW173045	MW446578	MW248744
*F. crassum*	CPC 37122	MW173061	MW446594	MW248760
*F. cuneirostrum*	NRRL 31157	EF408519	FJ240389	EF408414
*F. cyanescens*	NRRL 37625	EU329684	EU329637	FJ240353
*F. euwallaceae*	NRRL 54726	JQ038018	JQ038032	JQ038011
*F. falciforme*	CBS 47567	EU329690	LT960558	LT906669
*F. ferrugineum*	NRRL 32437	DQ094446	EU329581	DQ246979
*F. floridanum*	NRRL 62606	KC691561	KC691622	KC691533
*F. illudens*	NRRL 22090	AF178393	JX171601	AF178326
*F. keratoplasticum*	NRRL 46437	GU170643	GU170588	GU170623
*F. kuroshium*	UCR3641	KX262196	KX262256	KX262216
*F. lichenicola*	NRRL 28030	DQ094355	KR674002	KR673968
*F. metavorans*	NRRL 43489	DQ790528	DQ790572	DQ790484
*F. neocosmosporiellum*	NRRL 22166	DQ094319	EU329497	AF178350
*F. oblongum*	NRRL 28008	DQ094350	EF470135	DQ246868
*F. parceramosum*	CBS 115695	JX435199	JX435249	JX435149
*F. petroliphilum*	CBS 135955	KJ867425	KJ867426	KJ867424
*F. phaseoli*	NRRL 22276	EU329668	JX171608	EF408415
*F. protoensiforme*	NRRL 22178	AF178399	EU329498	AF178334
*F. pseudensiforme*	NRRL 46517	KC691584	KC691674	KC691555
*F. quercinum*	NRRL 22652	DQ094326	EU329518	DQ246841
*F. riograndense*	CMF 12570	KT186366	KX534003	KX534002
*F. solani*	SICAUCC 23-0086	OR921242	PP110509	PP110511
*F. solani*	SICAUCC 23-0087	OR921243	PP110510	PP110512
*F. solani*	NRRL 43468	EF453093	EF469980	EF452941
*F. solani*	NRRL 43474	EF453097	EF469984	EF452945
*F. solani-melongenae*	NRRL 22101	AF178398	MG282399	AF178333
*F. solani-melongenae*	NRRL 52699	JF740905	JF741108	JF740782
*F. solani-melongenae*	CBS 101573	KM231798	KM232365	KM231927
*F. suttonianum*	NRRL 32858	DQ094617	EU329630	DQ247163
*F. tonkinense*	NRRL 46676	GU250669	GU25073	GU250546
*F. tuaranense*	NRRL 46518	KC691571	KC691632	KC691543
*F. vanettenii*	NRRL 22278	DQ094309	EU329501	AF178337
*F. vanettenii*	NRRL 22820	DQ094310	EU329532	AF178355
*F. vanettenii*	CBS 123669	KM231796	KM232364	KM231925
*F. vanettenii*	NRRL 45880	EU329689	JX171655	FJ240352
*F. virguliforme*	NRRL 31041	AY220239	JX171643	AY220193
*F. waltergamsii*	NRRL 32323	DQ094420	EU329576	DQ246951
*F. yamamotoi*	NRRL 22277	AF178401	FJ240380	AF178336
*F. yamamotoi*	NRRL 22163	AF178394	EU329496	AF178328

### 2.3 Pathogenicity test

Based on Koch’s hypothesis, pathogenicity tests were performed on the pathogen using the method of Yang Z. L. et al. (2022). The test strain was the representative strain SICAUCC 22-0086, and the inoculated materials comprised *Bletilla striata* seedlings of 12 plants that were 2 years old, healthy, and of consistent size (provided by the Chengdu Academy of Agriculture and Forestry Sciences).

The wheat grains were soaked in water for 12 h, weighed and bagged in about 300 g. The grains were sterilized in an autoclave at 121°C for 30 min. After cooling under aseptic conditions about 10 mycelia discs of 9 mm in diameter were inoculated and mixed with wheat grains, while the control was inoculated with mycelia discs without mycelium. Incubate in an artificial incubator at 25°C for 15 days. When the mycelium was full or wrapped with wheat grains, it was ready to be used. Wheat grains with mycelia were evenly mixed with soil at a ratio of 1:40, and about 1 kg of the soil mixture was weighed and used to plant nine potted *Bletilla striata* seedlings. As a control, wheat grains without mycelia were mixed with soil in the same manner and used to plant three potted *B. striata* seedlings. Artificial climate incubator culture conditions were set to 25°C, 12/12 h light/dark, and a humidity of about 52%. Disease symptoms and severity were observed and recorded every 5 days. Three seedlings per treatment and the experiment was replicated three times (*n* = 9). After symptoms appeared, diseased root samples were taken for re-isolation and identification of the pathogen.

### 2.4 Effects of culture media on mycelial growth and sporulation

To explore the effects of ten diverse culture media on the mycelial growth and sporulation of representative strain SICAUCC 22-0086, a 9 mm activated colony was placed in the center of ten media. This fungus inoculated, respectively, on potato dextrose agar (PDA), potato saccharose agar (PSA), corn meal agar (CMA), czapek-dox medium (Czapek), chalmers agar modified (modified Richard), peberdy, modified frey medium (modified Fries), extract agar (MEA), oatmeal agar (OA) czapek doxsolution agar (CDA), synthetic low nutrient agar (SNA) and incubated under sterile conditions for 7 days at 25°C and 12/12 h light/dark conditions. The colony diameter was measured to assess mycelial growth, and the colony characteristics [including shape, color, edge features, texture (gloss), and elevation] were observed and recorded. Additionally, sterile water was dropped into each plate to form a spore suspension, and sporulation was then assessed using a hemocytometer plate. There were three tests per treatment and three replicates (*n* = 9) per test.

### 2.5 Effects of carbon and nitrogen sources On mycelial growth and sporulation

To explore the effects of various carbon and nitrogen sources on mycelial growth and sporulation (Song et al., 2020; Li et al., 2021), five carbon sources (glucose, sucrose, maltose, starch, and lactose, plus no carbon source as a control) and eight nitrogen sources (potassium nitrate, sodium nitrate, ammonium sulfate, ammonium nitrate, urea, peptone, beef paste, and yeast extract, plus no nitrogen source as a control) were tested. A 9 mm activated colony was placed in the center of the medium (using modified Richard medium as the basic medium) and incubated under sterile conditions for 7 days at 25°C and 12/12 h light/dark conditions. There were three tests per treatment and three replicates (*n* = 9) per test. Mycelial growth and sporulation were measured as described in section 2.4.

### 2.6 Effects of temperature, photoperiod, and pH on mycelial growth and sporulation

To explore the effect of temperature on mycelial growth and sporulation, a 9-mm activated colony was placed in the center of a PDA plate and incubated for 7 days at temperatures of 15°C, 20°C, 25°C, 30°C, and 35°C under aseptic and 12/12 h light/dark conditions, respectively. To explore the effect of the photoperiod on mycelial growth and sporulation, a 9 mm activated colony was placed in the center of a PDA plate and incubated under sterile conditions for 7 days at 25°C under full light (24 h light), full darkness (24 h dark), or alternating light and dark (12/12 h light/dark). To explore the effect of pH on mycelial growth and sporulation, a 9 mm activated colony was placed in the center of a PDA plate and incubated for 7 days at aseptic, 25°C, and 12/12 h light/dark conditions at pH 3, 4, 5, 6, 7, 8, and 9. There were three tests per treatment and three replicates (*n* = 9) per test. Mycelial growth and sporulation were measured as described in section 2.4.

### 2.7 Fungicide assays

The inhibition rate of seven fungicides (which are detailed in Table 3) was determined by the mycelial growth rate method (Xin et al., 2020). First, 1 × 10^4^ mg/L active ingredient solution was prepared and diluted to 10, 50, 100, 200, 400 and 800 μg/mL with sterile water and mixed with PDA medium (1:9 ratio). PDA medium without a fungicide was used as the blank control. A 9 mm activated colony of representative strain SICAUCC 22-0086 was placed in the center of each fungicide-containing/blank control PDA plate and cultured at 25°C for 7 days. There were five replicates per fungicide concentration. Mycelial growth and sporulation were measured as described in section 2.4.

**Table 3:** The fungicide name and source agent of the test agent.

Fungicide name	Active ingredient quality fraction/%	Type	Manufacturer
Chlorothalonil	75%	WP	Syngenta (Suzhou) Crop Protection Co., Ltd.
Difenoconazole	10%	WG	Syngenta (Suzhou) Crop Protection Co., Ltd.
Iprodione	50%	WP	Syngenta (Suzhou) Crop Protection Co., Ltd.
Mancozeb	70%	WP	Rohm and Haas Company
Pyraclostrobin	25%	EC	Hubei Maoerwo Biopharmaceutical Co., Ltd.
Thiophanate-Methyl	70%	WP	Shandong Zouping Pesticide Co., Ltd.
Triadimefon	15%	WP	Shenyang Sci Tech Chemical Co., Ltd.

EC, emulsifiable concentrate; WG, water-dispersible granules; WP, wettable powder.

The following formula was used to determine the antifungal rate of each fungicide at each concentration (where *D* is the control colony diameter and *d* is the treatment colony diameter):


$$\mathrm{Antifungalrate}\left(\%\right)=\frac{D-d}{D}\times 100\%$$

Microsoft Excel 2016 was used to calculate a toxicity regression equation (where *x* is the logarithm of the fungicide concentration (μg/mL) and y is the inhibition rate) and correlation coefficient for each fungicide. DPS v2.0 was used to calculate the half maximal effective concentration (EC_50_) value of each fungicide.

### 2.8 Optimization of fungicide concentration scheme using response surface methodology

The approximate ranges of antimicrobial rate of different agent concentrations were initially obtained through a one-way test, and the three fungicides with the highest inhibition rates were selected as the three factors in the response surface methodology (RSM) analysis using the Box–Behnken design, with antimicrobial rate (based on colony diameter) as the response value. From there, the factors and level values required for the orthogonal test were determined, and then the orthogonal test design was used to further optimize the optimal fungicide concentration scheme.

### 2.9 Data analysis

The data were analyzed using SPSS v24.0, and the ANOVA was done after conforming to the normal distribution test, means were analyzed for significance of differences using Duncan’s new complex polar method, and labeled using sequential letter labeling. Toxicity regression equations, correlation coefficients, and EC_50_ values were generated using Microsoft Excel 2016 and DPS v2.0. Graphs were constructed using GraphPad Prism v8. RSM was performed using Design-Expert v12.

## 3 Results

### 3.1 Disease symptom in the field

**Figure 1:**
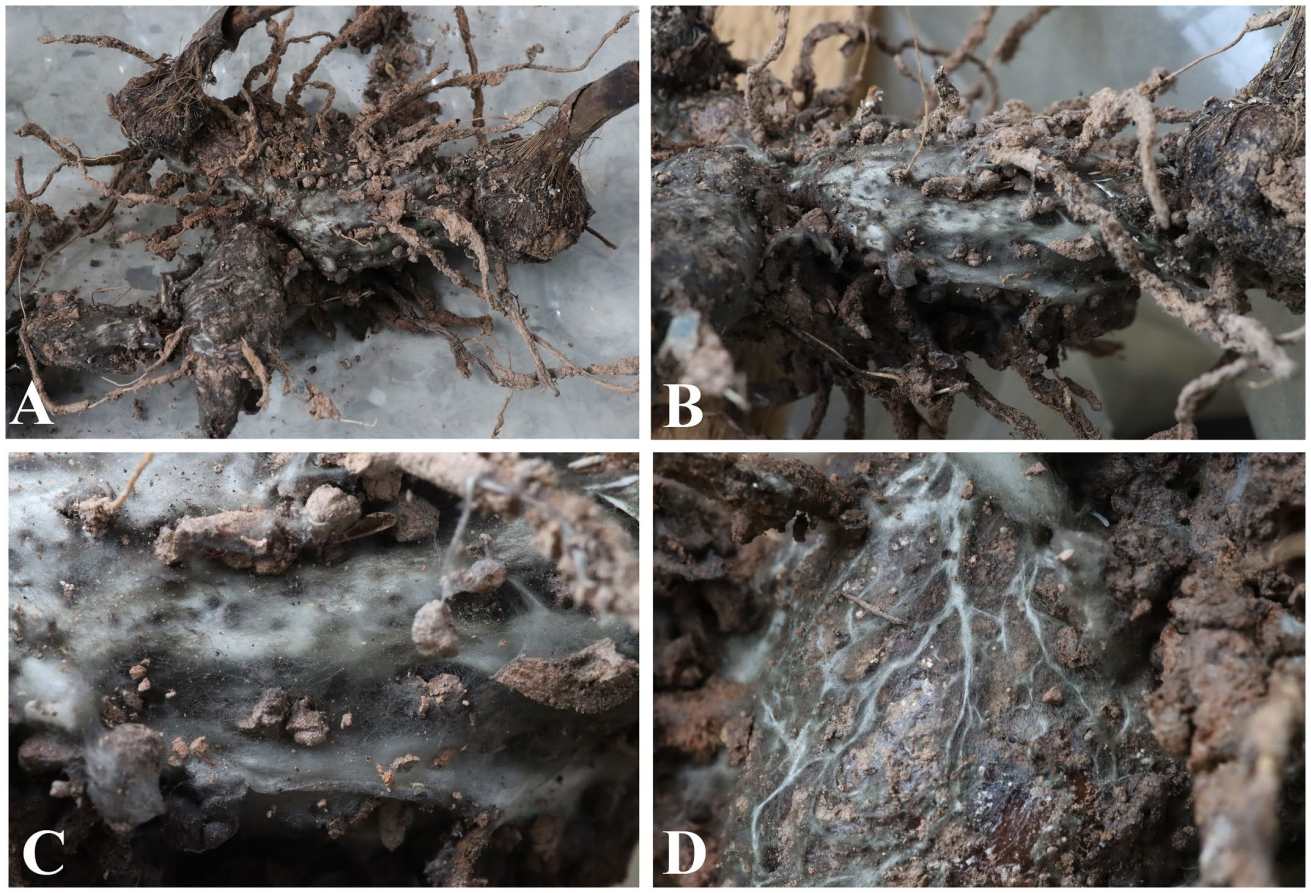
Disease symptoms on roots of *Bletilla striata* in the plant nurseries. **(A)** Whole root of *B. striata* showing severe necrotic symptoms. **(B–D)** Roots of *B. striata* covered by large amounts of white mycelia.

*Bletilla striata* plants with typical white root rot symptoms (Figure 1) were observed in plant nurseries of the Chengdu Academy of Agriculture and Forestry Sciences in Chengdu, Sichuan Province, China. All symptoms were observed in both plant nurseries surveyed. Field observation demonstrated that the underground roots had severe decay and necrosis. Their roots are covered with a mass of white filamentous to arachnoid mycelia. A white radial mycelia or rhizomorph develop on the old/main root, displaying a relatively loose and soft structure. The rhizomorph could expand into the soil, become thinner, and sometimes fill the gaps in the soil. The mycelia passed through the cortex and cambium, entering deep into the xylem, resulting in full root decay, leaf narrowing, gradual leaf yellowing, early leaf shedding and, ultimately, death of the entire plant. The incidence of *B. striata* white root rot was about 5%.

### 3.2 Morphological characteristics

Ten *Bletilla striata* samples with typical symptoms were collected from the two plant nurseries. We successfully isolated 10 *Fusarium* spp. (identified based on morphology) from the 10 diseased *B. striata* plants assessed.

**Figure 2:**
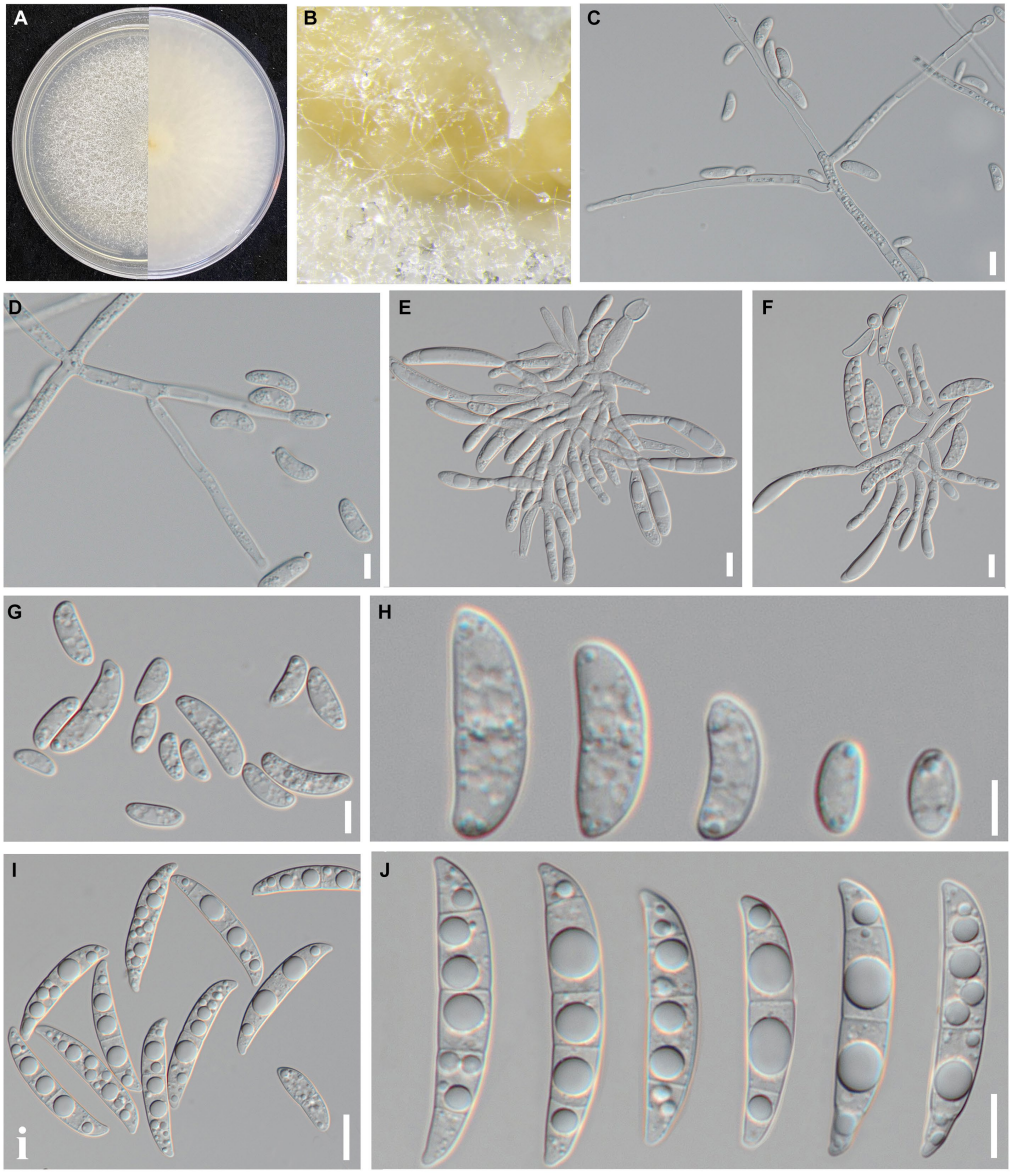
Culture and morphological characteristics of *Fusarium* sp. strain SICAUCC 23-0086. **(A)** Upper and reverse sides of colony on a PDA plate. **(B)** Conidia pile. **(C–F)** Conidiophores with developing conidia. **(G–H)** Microconidia. **(I–J)** Macroconidia. Scale bars: 20 μm **(C–F)**; 10 μm **(G–J)**.

Indoor culture characteristics: Colonies on PDA in the dark at 25°C reached a diameter of 70–75 mm after 7 days. Colonies regularly circular; reverse side white; filiform to arachnoid white mycelia (Figure 2). Sporulation from conidiophores formed on aerial mycelia. Conidiophores simple or branched one to several times, with each branch bearing a single terminal monophialide. Conidiogenous cells monophialidic, cylindrical, and could produce microconidia and macroconidia. Microconidia formed on aerial conidiophores, clustered in false heads at monophialide tips, and hyaline, oval or obovoid, symmetrical or gently bent dorsoventrally, smooth, and thin-walled, 0(−1) septate. 0-septate conidia: 9.07–18.22 × 3.83–8.26 μm (*x̅* = 15.6 × 6.3 μm, *n* = 50); 1-septate conidia: 23.14–37.52 × 7.83–10.26 μm (*x̅* = 29.6 × 9.3 μm, *n* = 50). Macroconidia hyaline, clavate to falcate, slight to moderate dorsal curvature, elongate and slender, 2–4 septate, predominantly 3 septate. 2-septate conidia: 48.26–53.74 × 8.59–9.52 μm (*x̅* = 49.5 × 9.2 μm, *n* = 50); 3-septate conidia: 50.14–58.36 × 8.75–11.33 μm (*x̅* = 52.6 × 10.2 μm, *n* = 50); 4-septate conidia: 55.78–62.04 × 9.05–11.5 μm (*x̅* = 57.2 × 9.3 μm, *n* = 50); contained oil drops, apical cells blunt or papillate, sporadically hooked, basal cells indistinct. Chlamydospores not observed.

Based on these morphological characteristics, the isolates matched the description of the genus *Fusarium* (Li et al., 2017; Crous et al., 2021; Han et al., 2023). Detailed morphological observation indicated that the 10 strains had completely consistent morphological characteristics with each other. To further validate this result, we selected two representative strains (SICAUCC 23-0086 and SICAUCC 23-0087) for phylogenetic analysis.

### 3.3 Molecular phylogeny

Molecular identification of two representative isolates (SICAUCC 23-0086 and SICAUCC 23-0087) was required to verify the accuracy of the morphological identification. Alignment against the NCBI GenBank database using BLASTn indicated that the ITS sequence from both strains shared 99.49% identity with that of *Fusarium solani* CBS 140079 with 100% query coverage, indicating that these strains could be a member of the *F. solani* species complex. Next, the *rpb*2 and *tef*1-α gene fragments were sequenced. The identity of each gene sequence (ITS, *rpb*2 and *tef*1-α) of the two isolates was 100%.

**Figure 3:**
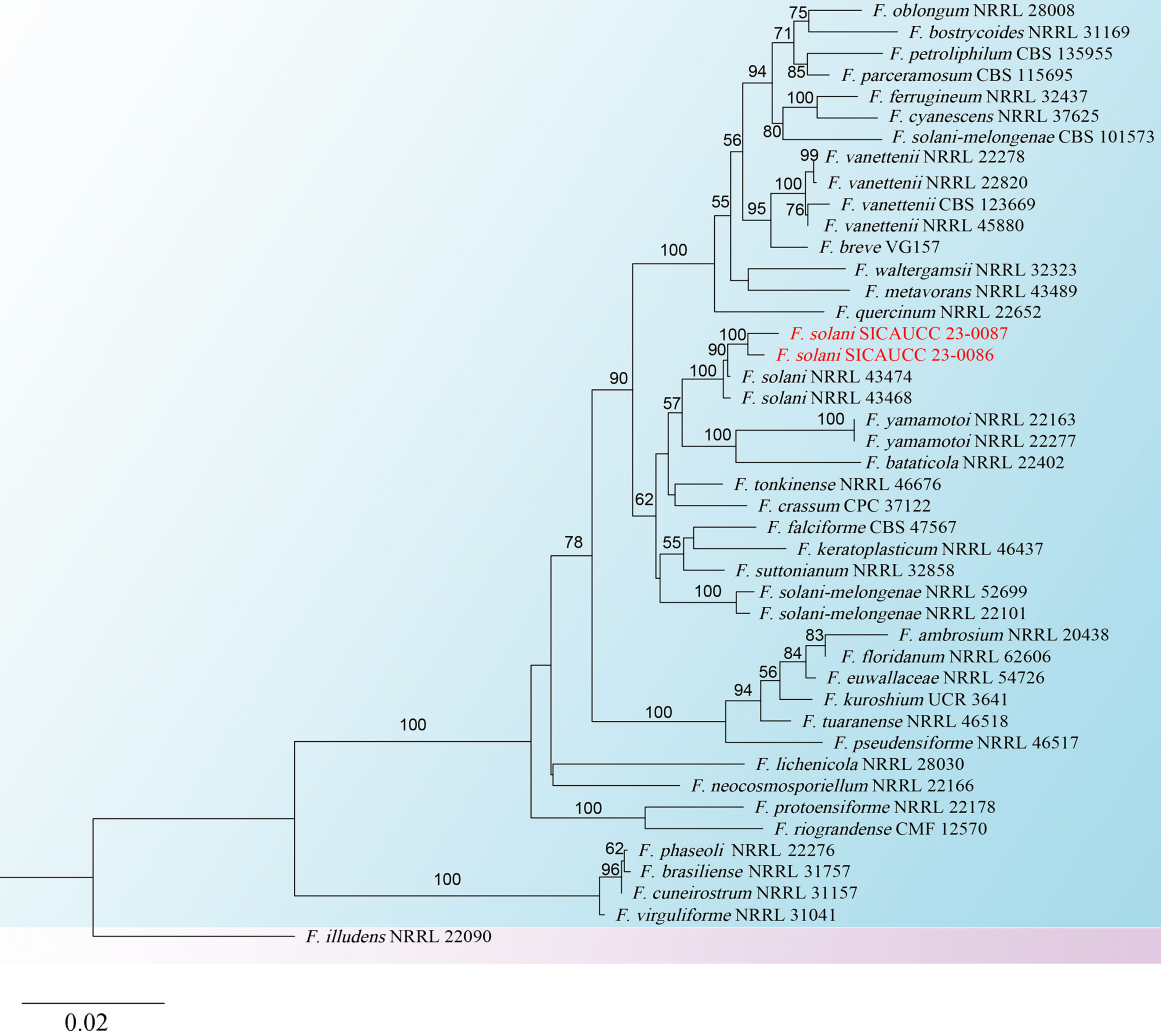
Phylogram generated from RAxML analysis, based on combined ITS, *rpb*2, and *tef*1-α sequence data of isolates. The best-scoring RAxML tree with a final likelihood value of −13876.220691 for ITS, *rpb*2, and *tef*1-α sequence data. The tree is rooted with *F. illudens* (NRRL 22090). The matrix had 1,020 distinct alignment patterns with 29.25% undetermined characters and gaps. Estimated base frequencies were as follows; A = 0.240624, C = 0.283489, G = 0.253665, T = 0.222222; substitution rates AC = 1.815518, AG = 5.297331, AT = 2.169463, CG = 1.145656, CT = 12.381417, GT = 1.000000; gamma distribution shape parameter *α* = 0.189753. Species identified in this study are indicated in red. Bootstrap support values (over 50%) from maximum likelihood are given at the nodes.

To identify the two isolates more accurately, a phylogenetic tree based on the concatenated sequences of ITS, *rpb*2, and *tef*1-a was constructed (Figure 3). *Fusarium illudens* NRRL 22090 was used as an outgroup and sequences from 47 taxa were included. The alignment contained 3,951 base pairs (ITS = 1,198, *rpb*2 = 2009, *tef*1-α = 744), including gaps. The phylogenetic tree showed that the two isolates clustered with *F. solani* NRRL43474 and *F. solani* NRRL43468. Bootstrap support values at the nodes were 100%. Therefore, the two isolates were identified as *F. solani* based on both morphological and molecular evidence.

### 3.4 Pathogenicity test

**Figure 4:**
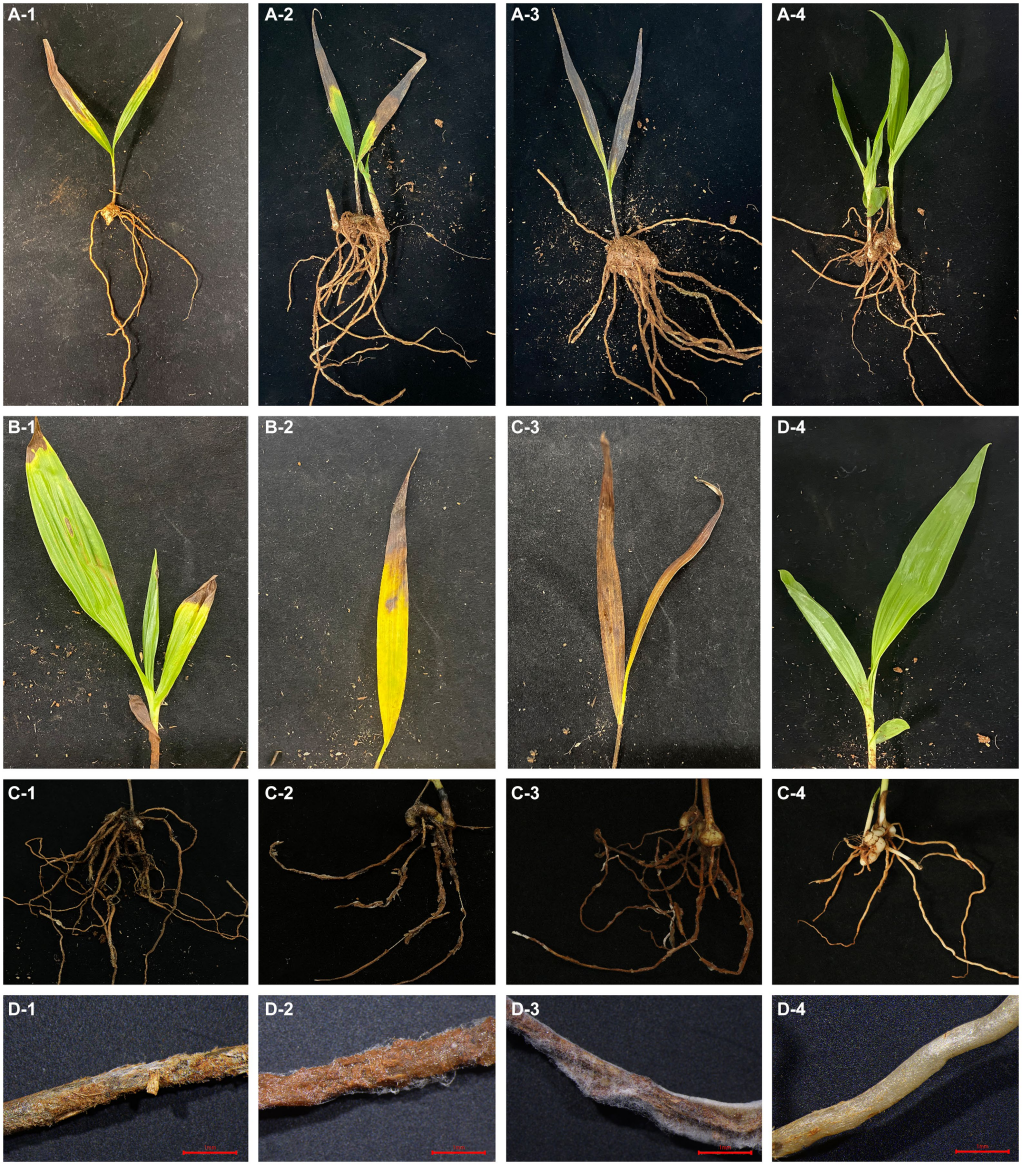
Phenotypes of *B. striata* seedlings at 5, 10, and 15 days post inoculation (dpi) with pathogen. The first, second, third, and fourth rows show the entire plant, leaves, roots, and root micrographs, respectively. **(A1–A3)** Infection status of entire plant at 5, 10, and 15 dpi. **(B1–B3)** Disease symptoms on leaves at 5, 10, and 15 dpi. **(C1–C3)** Disease symptoms on roots at 5, 10, and 15 dpi. **(D1–D3)** Micrographs of roots at 5,10, and 15 dpi showing decayed roots covered with mycelia. **(A4–D4)** Non-inoculated control plants with no symptoms on day 15. There were three pots per treatment and three seedlings (*n* = 9) per pot. Scale bars = 1 mm.

Mycelial discs of representative strain SICAUCC 22-0086 cultured for 7 days were used to inoculate wheat grains, which were then placed in the dark for 15 days. The mixture was added to soil, which was used to plant potted *Bletilla striata* seedlings (Figure 4). On day 5, the inoculated plants exhibited disease symptoms such as brown lesions, narrow leaves, and wilted tips (Figures 4A1,B1), similar to the symptoms observed in the field. There were a few white mycelia on the surface of the roots (Figures 4C1,D1). On day 10, the brown necrotic leaf lesions had gradually expanded, some leaves had withered or even shed (Figures 4A2,B2), the roots appeared rotten and were covered with white mycelia (Figures 4C2,D2), and the plant growth was significantly weakened. On day 15, all inoculated plants had wilting leaves (Figures 4A3,B3), the root epidermis and cortex were decaying, internal tissues were dark brown, and many white mycelia covered the rotting tissues (Figures 4C3,D3), similar to the symptoms observed in the field. In contrast, all non-inoculated plants remained healthy on day 15 (Figures 4A4–D4).

Next, diseased *Bletilla striata* seedling leaves and roots were randomly selected for pathogen re-isolation and identification. The re-isolated pathogens were consistent with the inoculated pathogens, fulfilling Koch’s postulates. Thus, we identified *Fusarium solani* as the pathogen causing white root rot in *B. striata*.

### 3.5 Optimum culture medium

**Figure 5:**
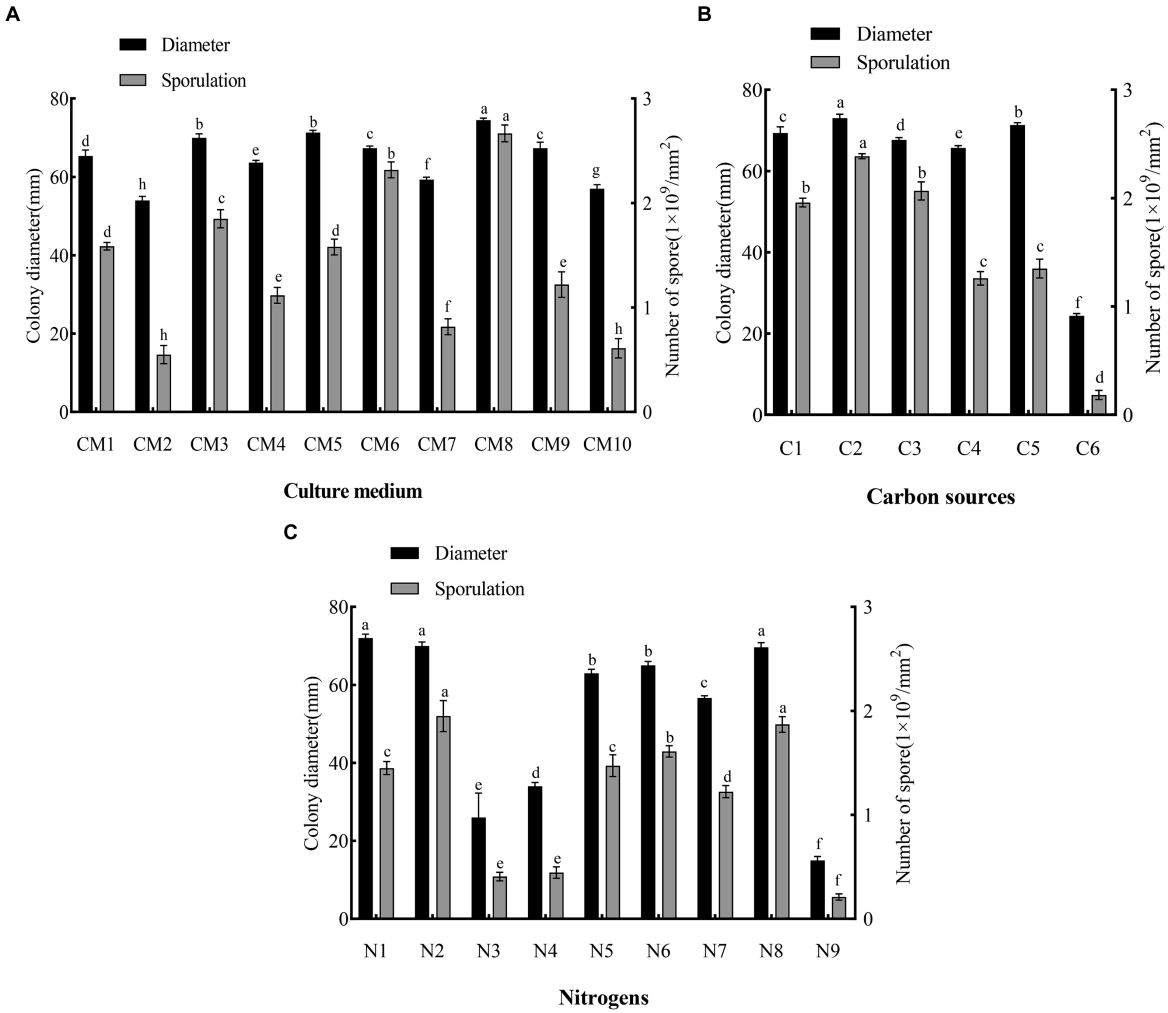
Effects of culture medium, carbon and nitrogen sources on mycelial growth and sporulation of *F. solani* SICAUCC23-0086. **(A)** Effect of different culture medium on mycelial growth and sporulation, where CM1 to CM10 are: PDA, PSA, CMA, Czapek, modified Richard, Peberdy, modified Fries, MEA, OA, and SNA medium. **(B)** Effect of different carbon sources on mycelial growth and sporulation. C1 to C6 are lactose, sucrose, maltose, starch, glucose, and no carbon source. **(C)** Effect of different nitrogen sources on mycelial growth and sporulation, N1 to N9 are potassium nitrate, sodium nitrate, ammonium sulfate, ammonium nitrate, urea, peptone, beef paste, and yeast extract were tested, and no nitrogen source. Different lowercase letters indicate significant differences (*p* < 0.05). Data are mean ± SD (*n* = 3).

The pathogen was able to grow and sporulate on all 10 media, but with significant differences in 7-day colony diameter and sporulation (Figure 5A). PDA was ranked first for growth (colony diameter: 74.5 mm, *p* < 0.05 vs. other treatments). Peberdy (colony diameter: 71 mm) and CMA (colony diameter: 69 mm) both ranked second (*p* > 0.05) for growth. MEA was ranked last for growth (colony diameter, 56 mm, *p* < 0.05 vs. other treatments).

PDA was ranked first for sporulation (2.74 × 10^9^ conidia/mm^2^, *p* < 0.05 vs. other treatments). OA was ranked second for sporulation (2.25 × 10^9^ conidia/mm^2^, *p* < 0.05 vs. other treatments). MEA (0.63 × 10^9^ conidia/mm^2^) and PSA (0.6 × 10^9^ conidia/mm^2^) were both ranked last (*p* > 0.05) for sporulation.

Based on both the colony diameter and sporulation, the optimum medium for the pathogen was PDA.

### 3.6 Optimum carbon and nitrogen sources

The pathogen grew and sporulated on the five carbon sources plus the control (Figure 5B). Sucrose was ranked first for both growth (colony diameter, 73 mm, *p* < 0.05 vs. other treatments) and sporulation (2.36 × 10^9^ conidia/mm^2^, *p* < 0.05 vs. other treatments). Glucose was good for growth (colony diameter, 71 mm, *p* < 0.05 vs. other treatments), but its effect on sporulation was middling (1.31 × 10^9^ conidia/mm^2^, *p* < 0.05 vs. other treatments). Control (no carbon source) was ranked last (colony diameter, 24 mm, *p* < 0.05 vs. other treatments; sporulation, 0.17 × 10^9^ conidia/mm^2^, *p* < 0.05 vs. other treatments). Overall, sucrose was the optimum carbon source.

The pathogen grew and sporulated on the eight nitrogen sources plus the control (Figure 5C). Potassium nitrate (colony diameter, 73 mm), yeast extract (colony diameter, 72 mm), and sodium nitrate (colony diameter, 69 mm) were all ranked first (*p* > 0.05) for growth. Sodium nitrate (2.1 × 10^9^ conidia/mm^2^) and yeast extract (1.95 × 10^9^ conidia/mm^2^) were ranked first (p > 0.05) for sporulation. Urea (colony diameter, 62 mm) and peptone (colony diameter, 65 mm) were both ranked second (*p* > 0.05) for growth. Control (no nitrogen source) was ranked last (colony diameter, 16 mm, *p* < 0.05 vs. other treatments; 0.21 × 10^9^ conidia/mm^2^, *p* < 0.05 vs. other treatments). Overall, sodium nitrate and yeast extract were the optimum nitrogen sources.

### 3.7 Optimum temperature, photoperiod, and pH

**Figure 6:**
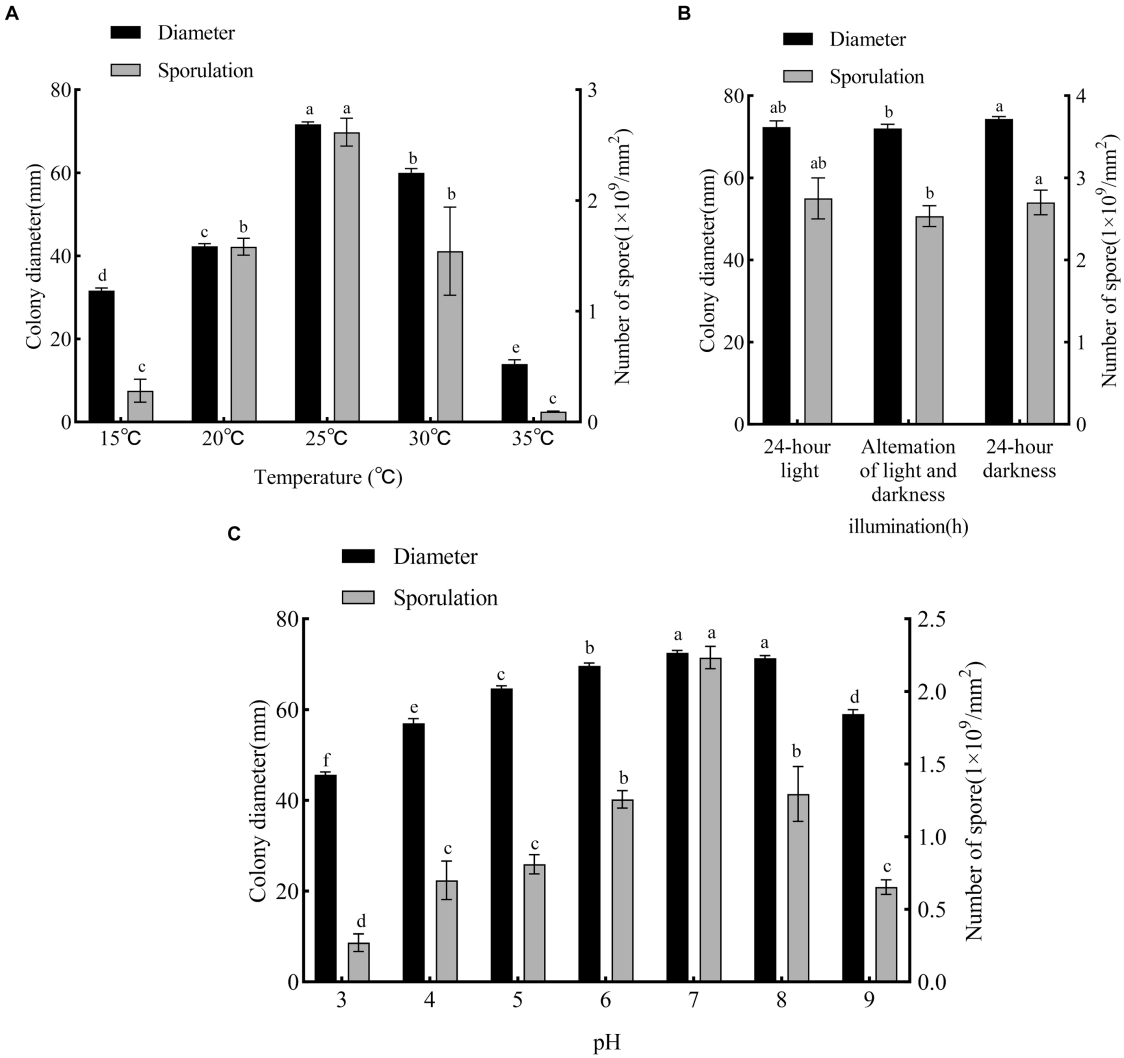
Effects of temperature, photoperiod and pH on mycelial growth and sporulation of *F. solani* SICAUCC23-0086. **(A)** Effect of different temperatures on mycelial growth and sporulation. **(B)** Effect of different photoperiods on mycelial growth and sporulation. **(C)** Effect of different pH on mycelial growth and sporulation. Different lowercase letters indicate significant differences (*p* < 0.05). Data are mean ± SD (*n* = 3).

The pathogen grew and sporulated at all temperatures tested (15–35°C), but with significant differences in colony diameter and sporulation (Figure 6A). At 25°C, growth was optimum (colony diameter, 72 mm, *p* < 0.05 vs. other treatments), as was sporulation (2.6 × 10^9^ conidia/mm^2^, *p* < 0.05 vs. other treatments). At 15°C (colony diameter, 31 mm; 0.2 × 10^9^ conidia/mm^2^) and 35°C (colony diameter, 14 mm; 0.09 × 10^9^ conidia/mm^2^), growth and sporulation were low. Therefore, 25°C was the optimum growth temperature, and lower and higher temperatures were unsuitable.

The pathogen grew and sporulated under all photoperiods tested (Figure 6B). Both 24 h light (colony diameter, 72 mm; 2.55 × 10^9^ conidia/mm^2^) and 24 h dark (colony diameter, 74 mm; 2.85 × 10^9^ conidia/mm^2^) were ranked first (*p* > 0.05) for both growth and sporulation. Overall, light vs. dark did not significantly affect growth or sporulation.

The pathogen grew and sporulated at all pH values tested (Figure 6C). At pH 7 and 8, growth was optimum (colony diameter, 73 and 71 mm, *p* < 0.05 vs. other treatments) and at pH 7 sporulation was optimum (2.37 × 10^9^ conidia/mm^2^, *p* < 0.05 vs. other treatments). Excessive acid and alkali were not conducive to growth or sporulation. Overall, the optimum pH was 7.

### 3.8 Fungicide assays

**Figure 7:**
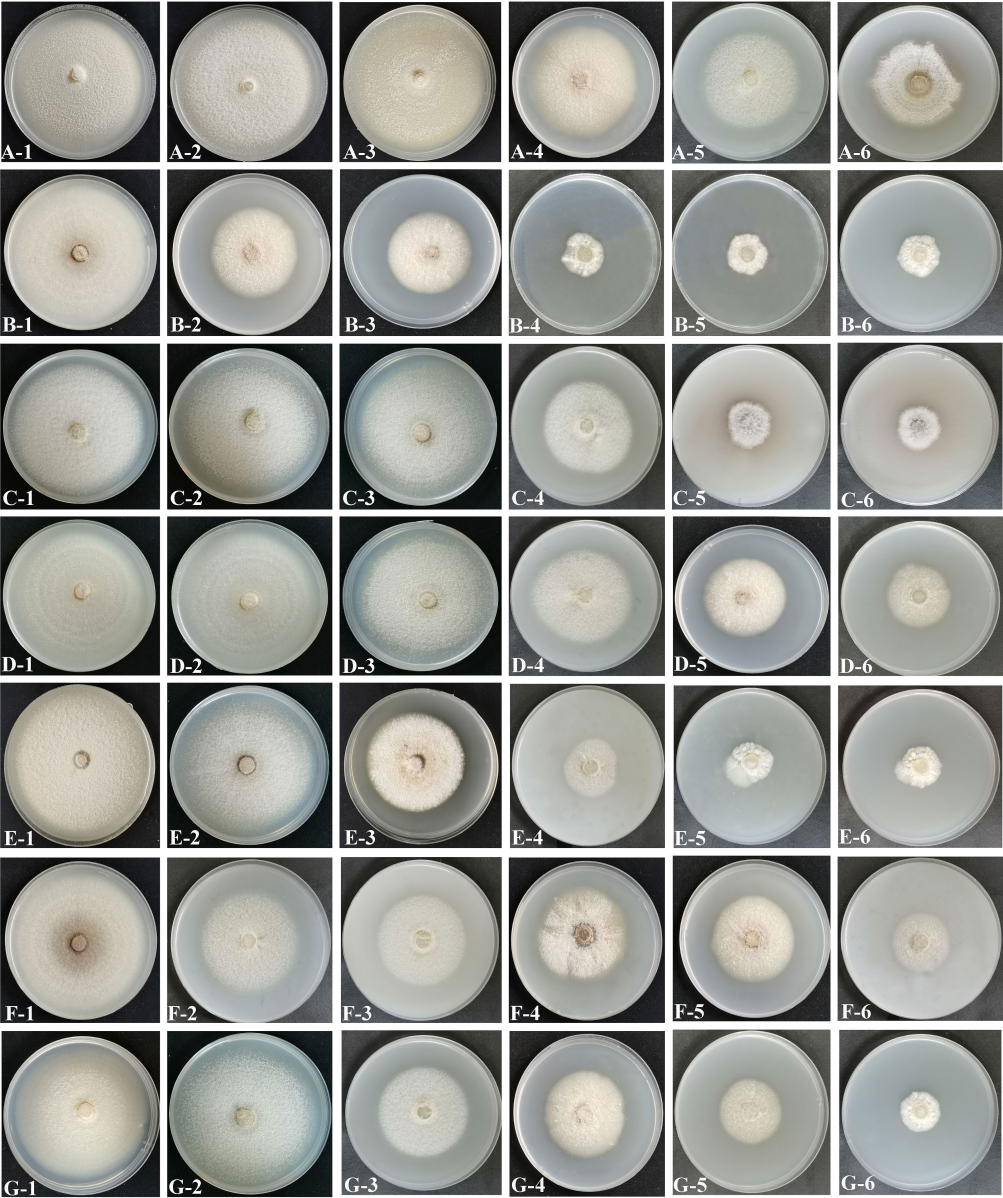
Effects of different fungicides on mycelial growth and spore production. **(A)** Chlorothalonil. **(B)** Difenoconazole. **(C)** Pyraclostrobin. **(D)** Mancozeb. **(E)** Thiophanate-Methyl. **(F)** Triadimefon. **(G)** Iprodione. 1–6, Corresponding to concentrations of 10 μg/mL, 50 μg/mL, 100 μg/mL, 200 μg/mL, 400 μg/mL and 800 μg/mL, respectively.

**Figure 8:**
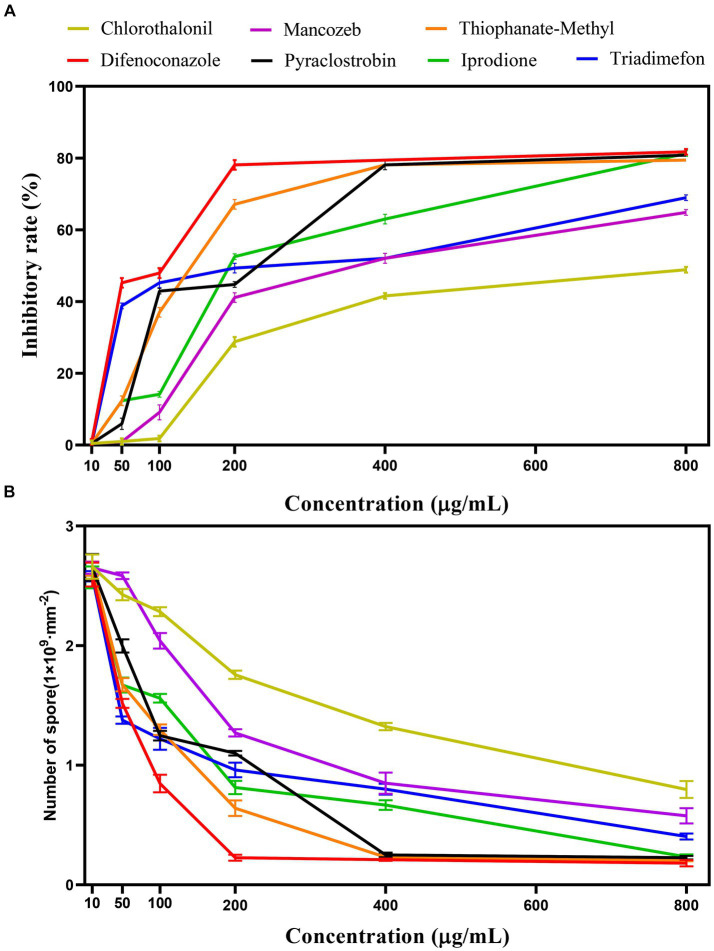
Effect of different fungicides on pathogen inhibition rate and sporulation. **(A)** Effect of different fungicides on the pathogen inhibition rate. **(B)** Effect of different fungicides on the sporulation.

Different fungicide types and concentrations led to different inhibition and sporulation rates of representative strain SICAUCC 22-0086 after culture on fungicide-containing PDA for 7 days (Figures 7, 8). At 200 μg/mL, difenoconazole had the highest inhibition rate (79.25%, *p* < 0.05 vs. other fungicides; sporulation, 0.23 × 10^9^ conidia/mm^2^). At 400 μg/mL, difenoconazole had the highest antifungal rate (80.11%; sporulation, 0.21 × 10^9^ conidia/mm^2^), followed by pyraclostrobin (78.18%; sporulation, 0.27 × 10^9^ conidia/mm^2^) and thiophanate-methyl (78.05%; sporulation, 0.24 × 10^9^ conidia/mm^2^) (*p* < 0.05 between these three vs. other fungicides). Chlorothalonil had the lowest inhibition rate (41.55%, *p* < 0.05 vs. other fungicides; sporulation, 1.33 × 10^9^ conidia/mm^2^). At 800 μg/mL, difenoconazole had the highest antifungal rate (81.74%; sporulation, 0.15 × 10^9^ conidia/mm^2^; *p* < 0.05 vs. other fungicides), followed by pyraclostrobin (80.82%; sporulation, 0.25 × 10^9^ conidia/mm^2^), thiophanate-methyl (80.03%; sporulation, 0.21 × 10^9^ conidia/mm^2^), and iprodione (79.45%; sporulation, 0.23 × 10^9^ conidia/mm^2^), *p* < 0.05 between these four vs. other fungicides. Chlorothalone had the lowest inhibition rate (48.86%, *p* < 0.05 vs. other fungicides; sporulation, 0.87 × 10^9^ conidia/mm^2^). In conclusion, among the seven fungicides tested, difenoconazole had the best inhibition rate, followed by pyraclostrobin and thiophanate-methyl, while chlorothalonil had the worst inhibition rate.

For each fungicide used in the fungicide assays, the original fungicide concentration and formulation type, toxicity regression equation (where *x* is the logarithm of the fungicide concentration (μg/mL) and y is the inhibition rate), correlation coefficient, and EC_50_ value are shown in Table 4. The best fungicide was 10% difenoconazole WG (EC_50_, 142.773 μg/mL), followed by 70% thiophanate-methyl WP (EC_50_, 183.924 μg/mL) and 25% pyraclostrobin EC (EC_50_, 235.593 μg/mL). 75% chlorothalonil WP was the worst (EC_50_, 693.091 μg/mL). In summary, difenoconazole, thiophanate-methyl, and pyraclostrobin effectively suppressed the colony diameter, with difenoconazole exhibiting the most profound impact.

**Table 4:** Toxicity of 7 fungicides against.

Fungicide name	Toxic regression equation	R	EC_50_ (μg/ mL)
10% Difenoconazole WG	*y* = 1.8151*x* + 1.0891	0.9209	142.773
15% Triadimefon WP	*y* = 1.6293*x* + 1.0293	0.9034	273.564
25% Pyraclostrobin EC	*y* = 1.9959*x* + 0.2654	0.9798	235.593
50% Iprodione WP	y = 1.8432x + 0.5463	0.9746	260.788
70% Mancozeb WP	*y* = 1.8074*x* + 0.2085	0.9107	447.760
70% Thiophanate-Methyl WP	*y* = 1.9502*x* + 0.5835	0.9563	183.924
75% Chlorothalonil WP	*y* = 1.5646*x* + 0.5553	0.9239	693.091

### 3.9 RSM

In the RSM analysis, the concentration factor levels of three agents, difenoconazole, pyrrologlucoside and thiophenate-methyl, were set based on the results of a one-way experiment. The inhibition rate was used as the response value to optimize these factor levels. Design-Expert v8.0.6 was used for RSM (including statistical analysis of variance, regression coefficients, and regression equations).

RSM yielded the following quadratic multinomial regression equation (where A is difenoconazole, B is pyraclostrobin, and C is thiophanate-methyl):


$$\begin{array}{l} Y=87.76+1.56A+0.8450B+1.86C-0.4275\mathrm{AB}\\ +\;0.1725\;\mathrm{AC}+0.5575\;BC-0.0487A^{2}+1.76B^{2}-9.65C^{2} \end{array}$$

The high *F*-value of the model (75.38) was highly significant (*p* < 0.001) (Table 5). C, A, and C^2^ had highly significant effects on inhibition rate (*p* ≤ 0.001), while B and B^2^ also significantly affected the inhibition rate (*p* < 0.05). Based on these results, the order of factors affecting the inhibition rate was as follows: thiophanate-methyl (C) > difenoconazole (A) > pyraclostrobin (B).

**Table 5:** Variance analysis of regression equation.

Source	Sum of square	df	Mean square	*F*-value	*p*-value	Significance
Model	454.53	9	50.50	75.38	<0.0001	***
A	19.53	1	19.53	29.15	0.0010	***
B	5.71	1	5.71	8.53	0.0223	*
C	27.75	1	27.75	41.42	0.0004	***
AB	0.7310	1	0.7310	1.09	0.3309	—
AC	0.1190	1	0.1190	0.1777	0.6860	—
BC	1.24	1	1.24	1.86	0.2153	—
A^2^	0.0100	1	0.0100	0.0149	0.9062	—
B^2^	13.02	1	13.02	19.44	0.0031	*
C^2^	392.20	1	392.20	585.39	<0.0001	***
Residual	4.69	7	0.6700	—	—	—
Lack of fit	3.27	3	1.09	3.06	0.1539	—
Pure rrror	1.42	4	0.3556	—	—	—
Cor. Total	459.22	16	50.50	—	—	—

The coefficient of determination (*R*^2^) was 0.9898 and the adjusted coefficient of determination (adjusted *R*^2^) was 0.9767 (Table 6), indicating that the model was sufficient to represent the relationship between the independent and response variables. Therefore, the model was statistically reasonable.

**Table 6:** Statistical parameters of the developed model.

Parameter symbol	Parameter name	Value
*R* ^2^	Coefficient of determination	0.9898
Adjusted *R*^2^	Adjusted coefficient of determination	0.9767
Predicted *R*^2^	Predicted coefficient of determination	0.8813
AP	Adeq precision	27.8721

**Figure 9:**
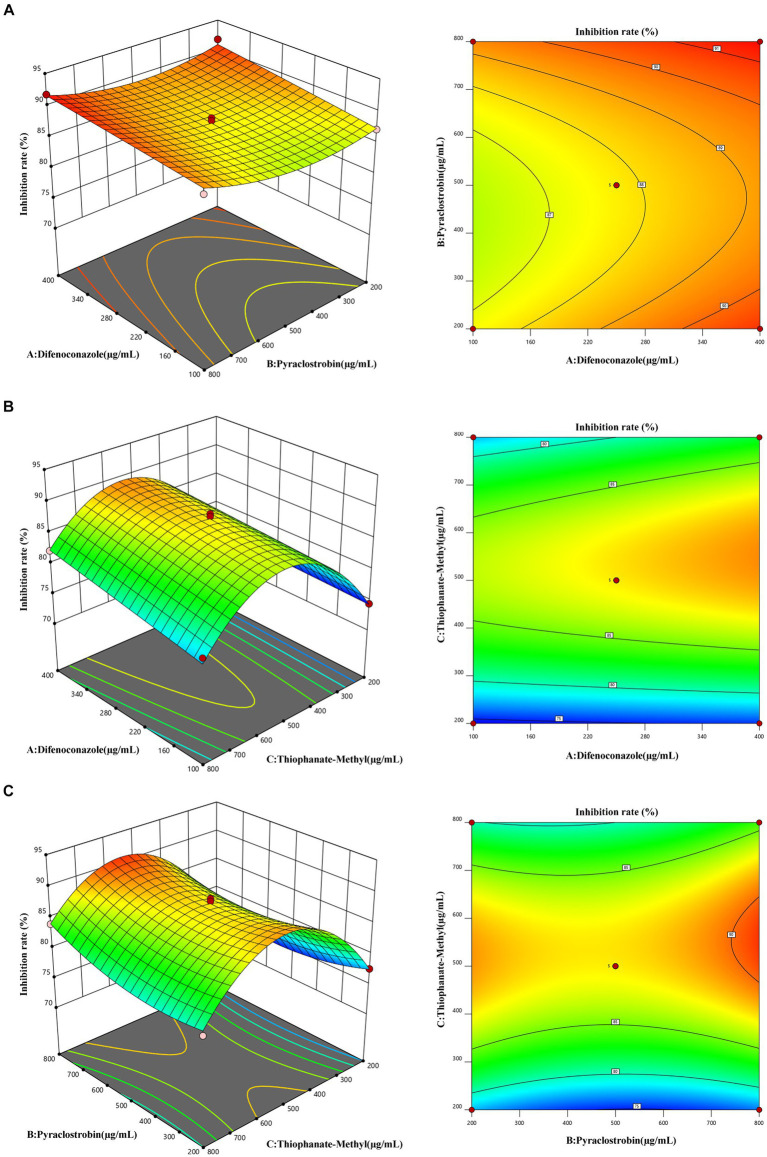
Response face interaction analysis. **(A)** Interaction between difenoconazole concentration and pyraclostrobin concentration. **(B)** Interaction between difenoconazole concentration and thiophanate-methyl concentration. **(C)** Interaction between pyraclostrobin concentration and thiophanate-methyl concentration.

The optimum fungicide concentration scheme for inhibition rate was determined by RSM (Figure 9). The optimum concentrations of difenoconazole, pyraclostrobin, and thiophanate-methyl were 395.42 μg/mL (Figure 8A), 781.03 μg/mL (Figure 8B), and 561.11 μg/mL (Figure 8C), respectively. Under this scheme, the inhibition rate was predicted to be 91.78 ± 0.18%. In practice, under this scheme, the inhibition rate was 92.24 ± 0.34% (based on three replicates), which was consistent with the prediction, thereby confirming the reliability of RSM.

## 4 Discussion

Root rot can be caused by various pathogens, including the species from *Fusarium*, *Pythium*, and *Phytophthora* (Ma, 2023). The pathogen first infects the root system, gradually weakening its water and nutrient absorption function. This leads to leaf yellowing and wilting, ultimately resulting in the death of the entire plant (Peña et al., 2013). Root rot is a major disease with a high incidence among various medicinal plants, including *Panax notoginseng*, *Atractylodes macrocephala koidz*, and *Astragali radix*, and it significantly impacts the yield and quality of the medicinal materials (Shen et al., 2014; Ma et al., 2019). The causal agents are typically soilborne fungi, with *Fusarium* species being particularly recognized as causal agents of root rot in numerous crops (Bodah, 2017; Yang K. et al., 2022).

*Bletilla striata* root rot has been reported to be caused by *Fusarium oxysporum* in Yunnan (Sun et al., 2013), *Epicoccum sorghinum* in Guilin, Guangxi (Ma, 2023), and *Rhizoctonia solani* in Guizhou (Zeng et al., 2012). Through pathogen isolation followed by morphological and molecular identification, we found that the pathogen causing *B. striata* white root rot in Chengdu, Sichuan Province, was *F. solani*, unlike in previous reports on *B. striata* root rot (Sun et al., 2013; Zhou et al., 2018; Li et al., 2019). *F. solani* has been reported to be the pathogen responsible for the development of leaf spot disease in *B. striata* (Zhou et al., 2020), which coincides with the symptoms we observed in the leaf of *B. striata*. It is noteworthy that there are similarities in some symptoms between this disease and previously reported root rot diseases of *B. striata* caused by *F. oxysporum, E. sorghinum*, and *Dactylonectria torresensis* (Sun et al., 2013; Zhou et al., 2018; Li et al., 2019). For example, at the beginning of the disease, brown leaf spots appeared on the above-ground parts, and as the disease progressed, the roots gradually became brown and softly rotted and severely decayed, resulting in the loss of water transport function of the roots, wilting of the leaves, and ultimately drying up to death. However, compared with the previous symptoms of *B. striata* root rot, *B. striata* root rot caused by *F. solani* shows a unique symptom on the decayed root tissues: the roots were covered with a large number of cobwebby white mycelia, which formed white radial mycelia or rhizomorph on the root surface and extended into the soil. These mycelia or rhizomorph can survive in the soil for many years, which undoubtedly increases the difficulty of controlling the root diseases of *B. striata*. Through the observation of disease symptoms, we found that the symptoms of *B. striata* disease are similar to those of white root rot of sand pear and white root rot of grapes (Zhu et al., 2012; Yang Z. L. et al., 2022; Wang et al., 2023), which mainly occurs in the roots, leading to drying and longitudinal cracking of diseased tissues and severe rotting. The surface of the diseased root is entangled with soft white mycelia, and these mycelia or rhizomorph are able to spread into the soil near the root bark, or spread on the soil surface at the base of the trunk. The pathogen mainly overwinters in the soil as mycelia or rhizomorph remaining on the diseased roots. In summary, we have identified *F. solani* as the pathogen that triggers *B. striata* white root rot. It has previously been reported that *F. solani* can also cause serious root rot in sweet potato, soybean, tobacco, tomato, and other economic crops (Luo et al., 2001; Romberg and Davis, 2007; Wang et al., 2014).

White root rot is widely distributed in China and is a relatively common soil-borne disease of roots. Since the disease is difficult to detect in the early stages due to root infection, by the time it is discovered, it has progressed and is therefore more difficult to treat. Usually, the host can only be removed in order to clear up the infection (Pal et al., 2020). In low-lying areas, water-logged soil with high viscosity and poor drainage can lead to poor plant growth, weak potential, and lead to increased disease. High temperature also favor the spread of disease (Wu, 2021). We assessed both environmental factors (such as photoperiod, pH, and temperature) and nutritional factors (such as carbon and nitrogen sources) for their effects on *Bletilla striata* white root rot. We found that the pathogen could grow and sporulate on all media tested, with PDA being the optimum medium. In addition, we found that all carbon and nitrogen sources supported mycelial growth and sporulation, but sucrose was the optimum carbon source, and yeast extract and sodium nitrate were the optimum nitrogen sources. The photoperiod did not significantly affect the mycelial growth and sporulation of the pathogen. Excessive acid and alkali were unfavorable for mycelial growth and sporulation, and the optimum pH for mycelial growth was pH 7. The optimum temperature for both mycelial growth and sporulation was 25°C. These results help us to understand *B. striata* white root rot occurrence and develop effective control measures.

Chemicals represent one of the main measures for the prevention and control of *Bletilla striata* pathogens (Dweba et al., 2017; Dai and Chen, 2020). We performed *in vitro* screening to identify fungicides that could effectively inhibit the growth of *Fusarium solani*. These fungicides will likely play an important role in future prevention and control practices to manage the spread of *F. solani* in order to combat *B. striata* white root rot. Among the seven pesticides, difenoconazole, thiophanate-methyl, and pyraclostrobin had strong inhibitory effects on pathogen growth, with difenoconazole exhibiting the best inhibition (EC_50_, 142.773 μg/mL). In addition, after conducting one-factor experiments, we used RSM to determine the optimum fungicide concentration scheme for maximizing the inhibition rate. According to RSM, the optimum fungicide concentration scheme comprised 395.42 μg/mL difenoconazole, 781.03 μg/mL pyraclostrobin, and 561.11 μg/mL thiophanate-methyl. Under this scheme, the inhibition rate was predicted to be 91.78 ± 0.18%. In practice, the inhibition rate was 92.24 ± 0.34%. In conclusion, RSM was used to determine the best fungicide concentration scheme to improve the inhibition rate, which provides a new strategy for combating the disease.

Overall, in this study, we found that *Fusarium solani* is the causal agent of *Bletilla striata* white root rot based on morphological, phylogenetic, and pathogenicity analyses. This is the first report of a causal agent of *B. striata* white root rot in China. The biological characteristics of the pathogen and the results of screening for prevention and control fungicides provide a basis for the comprehensive management of *B. striata* white root rot. This study presents a comprehensive analysis of the pathogens responsible for *B. striata* white root rot, encompassing pathogen identification, cultural omics characteristics, and screening for potential agents, thereby laying the groundwork for the development of control strategies.

## Data availability statement

The datasets presented in this study can be found in online repositories. The names of the repository/repositories and accession number(s) can be found in the article/supplementary material.

## Author contributions

FaL: Funding acquisition, Project administration, Conceptualization, Data curation, Formal analysis, Investigation, Methodology, Resources, Software, Supervision, Validation, Visualization, Writing – original draft, Writing – review & editing. XJ: Conceptualization, Formal analysis, Funding acquisition, Project administration, Resources, Software, Supervision, Visualization, Data curation, Investigation, Methodology, Validation, Writing – review & editing. LL: Funding acquisition, Project administration, Conceptualization, Data curation, Formal analysis, Investigation, Methodology, Resources, Software, Supervision, Validation, Visualization, Writing – review & editing. FW: Funding acquisition, Project administration, Resources, Conceptualization, Data curation, Formal analysis, Investigation, Methodology, Software, Supervision, Validation, Visualization, Writing – review & editing. FeL: Formal analysis, Funding acquisition, Project administration, Resources, Conceptualization, Data curation, Investigation, Methodology, Software, Supervision, Validation, Visualization, Writing – review & editing. SH: Data curation, Formal analysis, Funding acquisition, Methodology, Project administration, Resources, Supervision, Conceptualization, Investigation, Software, Validation, Visualization, Writing – review & editing. LT: Data curation, Formal analysis, Funding acquisition, Project administration, Resources, Supervision, Conceptualization, Investigation, Methodology, Software, Validation, Visualization, Writing – review & editing. XC: Data curation, Formal analysis, Funding acquisition, Methodology, Project administration, Resources, Supervision, Visualization, Conceptualization, Investigation, Software, Validation, Writing – review & editing. YX: Software, Validation, Writing – review & editing, Data curation, Formal analysis, Funding acquisition, Methodology, Project administration, Resources, Supervision, Visualization, Conceptualization, Investigation. XX: Conceptualization, Data curation, Investigation, Methodology, Resources, Software, Visualization, Writing – review & editing, Formal analysis, Funding acquisition, Project administration, Supervision, Validation. LJ: Conceptualization, Data curation, Investigation, Methodology, Resources, Software, Visualization, Writing – review & editing, Formal analysis, Funding acquisition, Project administration, Supervision, Validation. YL: Conceptualization, Data curation, Investigation, Software, Supervision, Validation, Writing – review & editing, Formal analysis, Funding acquisition, Methodology, Project administration, Resources, Visualization. CY: Conceptualization, Data curation, Formal analysis, Investigation, Methodology, Resources, Software, Supervision, Validation, Visualization, Writing – original draft, Writing – review & editing, Funding acquisition, Project administration.
